# Free amino acid and acylcarnitine values in *Ursus americanus* Pallas 1780 (black bear) from Northeastern Mexico

**DOI:** 10.1371/journal.pone.0272979

**Published:** 2023-02-03

**Authors:** Andres Abellan-Borja, Iram P. Rodriguez-Sanchez, Rogelio Carrera-Treviño, Olga Karina Villanueva-Segura, Patricio Adrian Zapata-Morin, Laura E. Martinez-de-Villareal, Luis Javier Barboza-Aranda, Mayra A. Gomez-Govea, Margarita L. Martinez-Fierro, Ivan Delgado-Enciso, Gabriel Ruiz-Ayma, Jose Ignacio Gonzalez-Rojas, Antonio Guzman-Velasco

**Affiliations:** 1 Facultad de Ciencias Biológicas, Laboratorio de Fisiología Molecular y Estructural, Universidad Autonoma de Nuevo Leon, San Nicolas de los Garza, Mexico; 2 Facultad de Medicina Veterinaria y Zootecnia, Laboratorio de Vida Silvestre, Universidad Autonoma de Nuevo Leon, General Escobedo, Mexico; 3 Departamento de Genetica, Facultad de Medicina, Universidad Autonoma de Nuevo Leon, Monterrey, Mexico; 4 Laboratorio de Medicina Molecular, Universidad Autonoma de Zacatecas, Zacatecas, Mexico; 5 Universidad de Colima, Colima, Mexico; 6 Secretaria de Salud de Colima, Instituto Estatal de Cancer, Colima, Mexico; 7 Facultad de Ciencias Biologicas, Laboratorio de Conservacion de Vida Silvestre y Desarrollo Sustentable, Universidad Autonoma de Nuevo Leon, San Nicolas de los Garza, Mexico; University of Veterinary Medicine Vienna: Veterinarmedizinische Universitat Wien, AUSTRIA

## Abstract

**Introduction:**

*Ursus americanus* Pallas 1780 is the largest carnivore and the only ursid in Mexico. It is considered an endangered species in the country because its distribution and population have been reduced by up to 80% because of habitat loss or furtive hunting. These problems can lead to a diet change, which could result in metabolic disorders, such as fatty acid β-oxidation defects or organic acid metabolism disorders. In our study, a free amino acid and acylcarnitine profile was characterized.

**Methods:**

Peripheral blood samples were drawn from nine free-ranging black bears in a period of five months, from June to October of 2019 in Northeastern Mexico, and 12 amino acids and 30 acylcarnitines were determined and quantified. Age differences were observed in the samples through ANOVA and *post-hoc* Tukey test.

**Results:**

Only three metabolites showed a significant difference with age: alanine (Ala) [cubs vs juvenile], free-carnitine (C0) [juvenile vs cubs] and acetylcarnitine (C2) [cubs vs adults and juvenile vs cubs].

**Conclusion:**

Metabolites with variability due to age were identified, making them potential biomarkers to monitor metabolic status as early diagnosis in endangered species. This is the first study of black bear amino acid and acylcarnitine profiles, and the values found could be used as reference for free amino acid and acylcarnitine concentrations in further studies of the species.

## Introduction

The black bear (*Ursus americanus* Pallas, 1780) is a medium-sized bear with distribution in North America [1]. The International Union for Conservation of Nature (IUCN) considers the species in the category of Least Concern (LC) at the global level [2]. In Mexico, the black bear is considered an endangered and protected species according to NOM-059-SEMARNAT-2010 [3]. There are 16 subspecies identified in North America of which three inhabit Mexico (*U*. *a*. *eremicus* Merriam, 1904, *U*. *a*. *amblyceps* Baird, 1859 and *U*. *a*. *machetes* Elliot, 1903). These are distributed in the states of Sonora, Chihuahua, Coahuila, Nuevo Leon, Tamaulipas, Durango, Zacatecas, San Luis Potosi, Sinaloa, Nayarit and Aguascalientes [1, 4–6] (see S1 Fig, available online). There have been reports of population reduction due to increased anthropogenic pressures on wildlife, habitat destruction (e.g., agriculture and livestock production), loss of natural prey/foods and rapid urbanization [4, 7–9].

Some health complications have been reflected in bears as a result of human-animal interaction such as physiological consequences of consuming low-energy foods could be a response to anthropogenic activity [10] or black bears alter movements in response to anthropogenic features with time of day and season [11]. Furthermore, *U*. *americanus* can change its natural behavior by consuming food from anthropogenic sources, during hyperphagia period which is before wintering [12]. Numerous metabolic changes occur in certain physiological states in bears such as hibernation, during which time there is leucine synthesis, reduced glucose use and increased lipolysis [1]. Also, bears come into contact with other free-ranging animals, which could transmit pathogens that can cause changes in the bears’ health [13]. An effective way of identifying metabolic disorders is by exploring free amino acids and acylcarnitine metabolites in blood using multianalyte technologies, such as tandem mass spectrometry (MS/MS) [14, 15]. The metabolic profile detects alterations in particular metabolic pathways, such as β-oxidation and organic acid metabolism disorders [16]. Free amino acid and acylcarnitine profiles have been used with different approaches to establish reference ranges in other organisms such as mosquitoes, rats, seals and horses [14, 16, 17]. A few studies in bear species such as *U*. *arctos* L (brown bear), have examined metabolite profiles, including lipids, bile acids, cortisol metabolites, acylcarnitines, amino acids, hydrogen sulfide and nitric oxide [18–21]. Metabolic profiles and the possible influence of exposure to pollutants have also been used in free-ranging polar bears (*Ursus maritimus* Phipps). In other hand, plasma amino acids and other metabolites like from Lipid and nitrogen have been studied in *U*. *americanus* during lactation [22–24]. Thus, it is hypothesized that the amino acid and acylcarnitine concentrations in the blood of black bear populations will differ. The aim of the present study was to establish free amino acid and acylcarnitine reference profiles through tandem mass spectrometry (MS/MS) [25, 26]. Learning about metabolic changes in an animal population is important to assess overall health, as well as determining possible infectious agents or metabolic disorders to understand the physiological response of wildlife to their environment. This is the first set of data on a free amino acid and acylcarnitine profile in black bears, making metabolomics potential tool for monitoring bear health.

## Material and methods

### Study area

**Fig 1:**
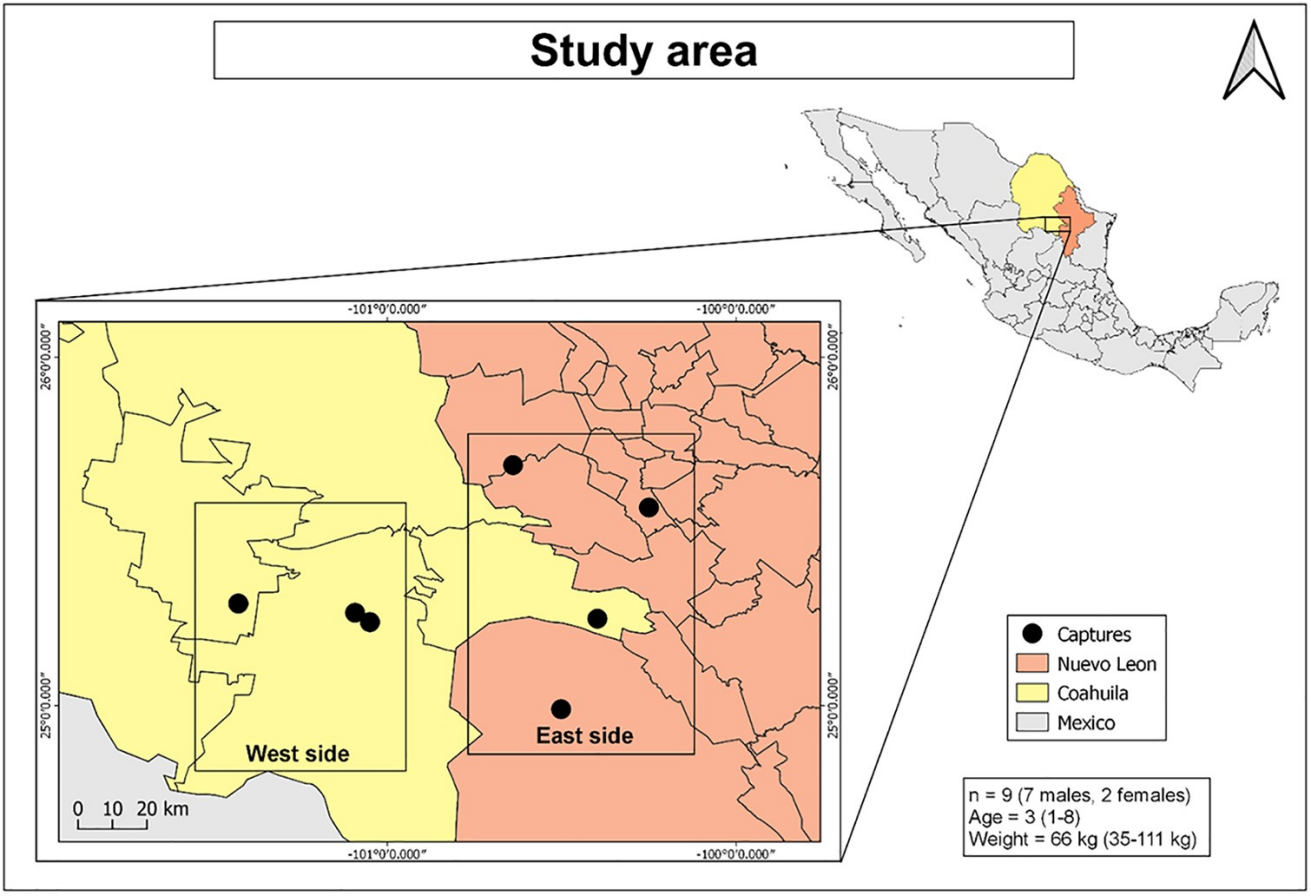
Study area. Coahuila (yellow) and Nuevo Leon (orange) states. In the lower right corner, information about the study population: sample size (n), median and range of age and weight. The figure was made in QGIS 3.10.10 suite (with CC BY 4.0 license) and loaded with INEGI (Instituto Nacional de Estadística y Geografía).

The study area was the Northeastern zone of the geological province Sierra Madre Oriental (SMO) located in the states of Coahuila and Nuevo Leon (Mexico) (Fig 1) [27]. It is part of the drainage basin of the Bravo-Conchos River. The elevation ranges from 600 to 3,400 meters above sea level (m.a.s.l.). The vegetation is in accordance with the altitude: at the lowest elevation, there is microphyllous, rosetophile, “chaparral” and submontane shrub, and the middle elevation is predominated by oak forests and oak-pine forests, while the highest elevation is represented by coniferous forests [28].

### Sample collection

Blood samples were collected from June to October 2019 from nine free-ranging black bears in the states of Nuevo Leon and Coahuila, Mexico (7 ♂, 2 ♀; age range 1–8 years; weight range 35–111 kg). These black bears were captured because they came into conflict or contact with humans. All methods were carried out in accordance with regulations of the General Agency of Wildlife (DGVS, in Spanish) based on standard NOM-059-SEMARNAT-2010 concerned with Environmental Protection of Native Species of Mexico. DGVS authorized the collection and sampling of the specimens with permission No. SGPA/DGVS/002160/18. The capture procedure and sample collection consisted of placing bait (sardines) inside a Cambrian-type trap. To avoid injury to the bear during transport and handling, it was anesthetized with zolazepam [Zoletil^®^ 100 (a combination of 50 mg/ml tiletamine and 50 mg/ml zolazepam, Virbac), Carros, France], which was administered with a pole syringe. The injected volume was based on weight and morphological size following the recommended doses (5 mg/kg) [29]. After the bear was anesthetized, respiratory rate, heart rate, body temperature and oxygen saturation were recorded every 10 minutes to ensure that these parameters were within the normal range [30]. Data including sex, age, weight, color and general measures were recorded during management (see S1 Table, available online). From the specimens, 3 mL of blood were collected in EDTA tubes (BD Vacutainer) from the femoral vein with a hypodermic needle.

After management, the bears were relocated to a nearby area where they could not cause problems with humans. Subsequently, blood samples were transported in a cooler with a refrigerant at 4°C to the Laboratorio de Fisiologia Molecular y Estructural at the Facultad de Ciencias Biologicas of the Universidad Autonoma de Nuevo Leon, where the samples were processed immediately upon arriving at the laboratory. The experimental protocols used in our study were approved by the Biosafety Committee of the Facultad de Medicina Veterinaria y Zootecnia of Universidad Autonoma de Nuevo Leon.

### Metabolomic analysis

Metabolomic analysis was done following a previously described procedure [15]. Each sample was analyzed to determine 12 amino acids and 31 acylcarnitines (see Table 1). A NeoBase non-derivatized MS/MS kit (PerkinElmer) was used to obtain the metabolites values, following the manufacturer’s instructions [31]. A solution included in the kit containing internal standards labeled with stable isotopes was used to quantify the metabolites of interest. The samples were analyzed in a mass spectrometer (TQD Waters). Sample analysis was performed with multiple-reaction monitoring using Maslynx Software.

**Table 1:** Amino acid and acylcarnitine levels determined in blood samples of free-ranging *U*. *americanus* Pallas, 1780. The column on the left lists the abbreviations of the metabolites analyzed. Mean, standard deviation (SD) and range (minimum and maximum) are shown.

Metabolite	Mean and SD (μmol/L)	Min (μmol/L)	Max (μmol/L)	Metabolite	Mean and SD (μmol/L)	Min (μmol/L)	Max (μmol/L)
Ala	307.569 ± 160.959	138.880	610.027	C6	0.011 ± 0.002	0.010	0.015
Arg	99.912 ± 24.24	57.485	124.693	C6DC	0.049 ± 0.009	0.040	0.067
Cit	37.334 ± 10.749	21.710	53.542	C8	0.012 ± 0.003	0.010	0.020
Gly	315.698 ± 111.128	139.520	457.193	C8:1	0.007 ± 0.004	0.000	0.013
Leu	144.661 ± 37.241	72.370	213.620	C10	0.009 ± 0.002	0.005	0.010
Met	6.955 ± 2.999	2.660	12.237	C10:1	0.004 ± 0.004	0.000	0.010
Orn	46.234 ± 14.053	16.363	62.977	C10:2	0.003 ± 0.003	0.000	0.010
Phe	55.47 ± 11.965	37.407	75.233	C12	0.01 ± 0.001	0.008	0.010
Pro	93.196 ± 29.411	44.710	136.157	C12:1	0.009 ± 0.001	0.007	0.010
Tyr	37.241 ± 8.268	22.250	52.770	(C14)	0.034 ± 0.012	0.010	0.047
Val	119.681 ± 38.136	50.940	190.400	Tetradecenoylcarnitine (C14:1)	0.018 ± 0.004	0.010	0.023
SA	0.323 ± 0.042	0.255	0.405	Tetradecadienoylcarnitine (C14:2)	0.008 ± 0.002	0.003	0.010
C0	10.888 ± 3.838	5.920	16.588	Hexadecanoylcarnitine (C16)	0.306 ± 0.135	0.058	0.560
C2	4.572 ± 1.886	2.483	8.792	Hexadecenoylcarnitine (C16:1)	0.053 ± 0.024	0.013	0.097
C3	0.229 ± 0.072	0.143	0.373	3-OH-hexadecenoylcarnitine (C16:1OH)	0.028 ± 0.014	0.010	0.053
C3DC+C4OH	0.028 ± 0.01	0.017	0.050	3-OH-hexadecanoylcarnitine (C16OH)	0.011 ± 0.002	0.010	0.013
C4	0.352 ± 0.133	0.193	0.567	Octadecanoylcarnitine (C18)	0.115 ± 0.042	0.068	0.220
C4DC+C5OH	0.178 ± 0.023	0.150	0.220	Octadecenoylcarnitine (C18:1)	0.325 ± 0.084	0.173	0.43
C5	0.091 ± 0.028	0.057	0.137	3-OH-octadecenoylcarnitine (C18:1OH)	0.013 ± 0.004	0.01
C5:1	0.007 ± 0.003	0.003	0.010	3-OH-octadecadienoylcarn (C18:2)	0.111 ± 0.057	0.040	0.237
C5DC+C6OH	0.067 ± 0.028	0.045	0.140	3-OH-octadecanoylcarnitine (C18OH)	0.005 ± 0.003	0.002	0.010

### Statistical analysis

Data analysis was performed using R (https://www.r-project.org/) [32]. Raw measurements were normalized by sum of all values within a sample to 1, and data were then obtained by dividing each value by the sum of all values per sample. These normalized values were then converted to log base 2 to handle extreme values. A *t* test was used to determine statistically significant differences between groups. Age was divided into cub with 3 individuals (1–2 years), juvenile with 4 individuals (3–4 years) and adult with 2 individuals (8 years) [33], and ANOVA (*p* ≤0.05) and *post-hoc* Tukey test were performed. The heat maps are a graphic representation of the proportion of amino acids and acylcarnitines in the study groups. The heat maps were generated in the ggplot2 package of R software [34].

## Results

A total of 42 metabolites were analyzed, 12 amino acids and 30 acylcarnitines. Reference ranges were also established in peripheral blood in free-ranging black bears. Means, standard deviations and ranges of 42 metabolites are shown in Table 1. Amino acids with low concentrations were succinylacetone (SA) and methionine (Met) with values of 0.323 μmol/L (range 0.255–0.405 μmol/L) and 6.955 μmol/L (range 2.660–12.237 μmol/L), respectively; acylcarnitines with low concentrations were decenoyl-carnitine (C10:1) and decadienoyl-carnitine (C10:2) with values of 0.004 μmol/L (range 0.000–0.010 μmol/L) and 0.003 μmol/L (range 0.000–0.010 μmol/L). respectively. The amino acids with the highest concentrations were alanine (Ala) and glycine (Gly) at 307.569 μmol/L (range de 138.880–610.027μmol/L) and 315.698 μmol/L (range 139.520–457.193 μmol/L), and the acylcarnitines with the highest concentration were free carnitine (C0) and acetylcarnitine (C2) at 10.888 μmol/L (range 5.920–16.588 μmol/L) and 4.572 μmol/L (range 2.483–8.792 μmol/L), respectively.

**Fig 2:**
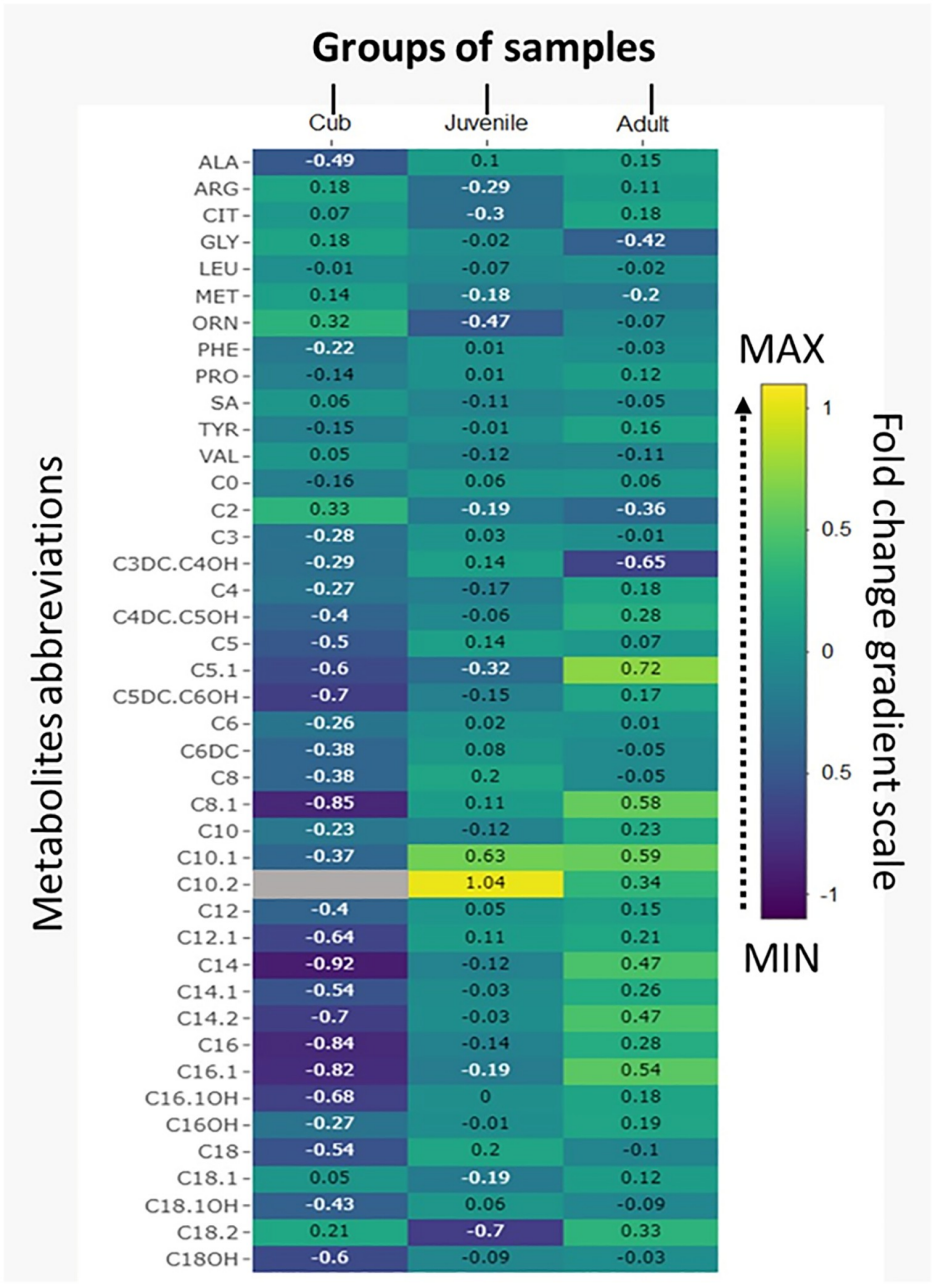
Heat map represents metabolites analyzed by folds in cubs (0–2 years), juveniles (3–6 years); adults (≥7 years).

Heat maps of the 42 metabolites by folds are represented in Fig 2, distinguished by age: cubs (0–2 years), juveniles (3–6 years), adults (≥7 years). The yellow, green and purple colors mean high, medium and low metabolite concentrations. C10.2 showed the highest concentration in juvenile bears. ANOVA performed between different age groups of bears showed that there was a significant difference only in three metabolites, alanine (Ala), free carnitine (C0) and acetylcarnitine (C2) (Table 2).

**Table 2:** Significant differences in amino acid and acylcarnitine reference values in blood samples of free-ranging *U*. *americanus* Pallas. Comparative analysis between different ages of bears. The column on the left gives the abbreviations of the metabolites analyzed; comparisons between cub (1–2 years), juvenile (3–4 years) and adult (8 years) bears are shown. A significant difference was indicated by *p* <0.05.

Metabolite	Age	
	Comparison	*p*-value
**Ala**	Cub vs juvenile	0.0096
**C0**	Juvenile vs cub	0.0267
**C2**	Cub vs adult	0.0001
	Juvenile vs cub	0.0016

## Discussion

Blood acylcarnitines are usually used to determine aberrations in mitochondrial β-oxidation of fatty acids, although little has been done in the determination of reference values in bears at different ages. Also, metabolite profiles determined by tandem MS have become a useful tool for detecting illnesses that have nonspecific signs and symptoms, and thus, an acylcarnitine profile can be beneficial in identifying fatty acid oxidation defects [15, 26]. Metabolite profiles in free-ranging bears have proven to be a powerful tool in detecting changes, such as in identifying the potential influence of contaminant exposure in two subpopulations of polar bears (*U*. *maritimus* Phipps) through isotope ratio mass spectrometry (IRMS) [22]. Another example of such was the determination of seasonal metabolic changes through the active phase and hibernation in the brown bear (*U*. *arctos* L), also using MS/MS as in the present study [20]; other techniques such as high-performance liquid chromatography (HPLC) have also been used in this type of study in bears [18]. It is relevant that although the Mexican black bear is an endangered species [3], no metabolomic studies have been done to establish a metabolic status. This is the first metabolite dataset (amino acids and acylcarnitine) determined in a Mexican *Ursus* species, and thus, means and SD of free amino acids and acylcarnitines in free-ranging black bears are reported (Table 1) for comparisons in further analyses with the same species, as it is believed that habitat loss, human population and trash in the suburbs will increase in the near future. Therefore, this is likely to affect the black bears’ diet [6] and can be reflected in this kind of analysis [35].

Acylcarnitine and amino acid levels have been reported in metabolic studies in humans; values are influenced by sex, age, diet and status at the moment of sample collection [17, 35–37]. We compared bear groups to examine differences by age. Studies in horses and humans showed significant differences between different ages [9, 38]. Similarly, we found here a significant difference in alanine (Ala), glycine (Gly) and acetylcarnitine (C2). This can be associated with differences in diet during the life of the bear, which is reflected in the amino acid and acylcarnitine profile [39–42]. Likewise, a significant difference was observed in three metabolites between ages, namely Ala, free carnitine (C0) and C2, especially in early ages from 1 to 2 years with respect to the others (3 to 4 and 8 years). Our results are like previous findings on mammals already mentioned. It is important to point out that decadienoyl carnitine (C10:2) was not detectable in 1- to 2-year-old bears (Fig 2); in humans, this acylcarnitine, when increased, is usually related to DCR 24-dienoyl-CoA reductase deficiency [37].

These findings can be useful to relate metabolites such as Ala, C0 and C2 to diet variations and can be the basis for detecting diet changes in the future. Amino acids play an important role in regulating food intake and nutrient metabolism in mammals [14]. Ala inhibits pyruvate kinase; therefore, it is responsible for regulating gluconeogenesis and glycolysis to ensure net glucose production during periods of food deprivation [43]. It has been proposed to be a nutritionally essential amino acid based on dietary intake of glycine and its need [44]. Individual acylcarnitines may imply changes in specific metabolic pathways, such as physiological and genetic mechanisms. In addition, regarding variation due to location, the variation factor in Ala, C0 and C2 can be referred to age in further studies. C2 is an acetylated form of carnitine that is synthetized naturally intra-mitochondrially, and it is involved in trans-mitochondrial membrane trafficking of acetyl units in many pathways, including glycogen, lipid and acetylcholine synthesis [45]. C2 increase is the result of β-oxidation because of fitness cost [16, 46], or if low, it can be related to a ketosis condition [36]. C0 is essential for the transfer of long-chain fatty acids through the inner mitochondrial membrane for β-oxidation, and it is known that in mammals, low levels of C0 are normally associated with primary carnitine deficiency [47]. It is important to mention that it is known that the blood concentration of these kinds of metabolites are regulated and stable in mammals, such as rats and humans [48]. Although a change in the concentration of the metabolites observed may be due to the change in diet during the different stages of the bear, subsequent studies should be carried out on their effect on metabolism.

In conclusion, free amino acid and acylcarnitine ranges were established for the first time in free-ranging black bears. Metabolites with variability due to diet and age were identified, making them potential biomarkers to monitor metabolic status for early diagnostic purposes in endangered species. Future comparisons with the ranges of amino acids and acylcarnitine established here, could lead to management measures. This is the first metabolomic analysis in this species that is a useful way for health monitoring in this and other endangered species. It is important to increase sample numbers to identify those metabolites that vary due to a change in diet that could affect a species metabolic status.

## Supporting information

S1 Fig (TIF)

S1 Table (XLSX)
