# Supplementary figures and images for: Free amino acid and acylcarnitine values in Ursus americanus Pallas 1780 (black bear) from Northeastern Mexico
Source: PLoS One. 2023 Feb 3;18(2):e0272979. doi: 10.1371/journal.pone.0272979 (PMC9897576; doi:10.1371/journal.pone.0272979)

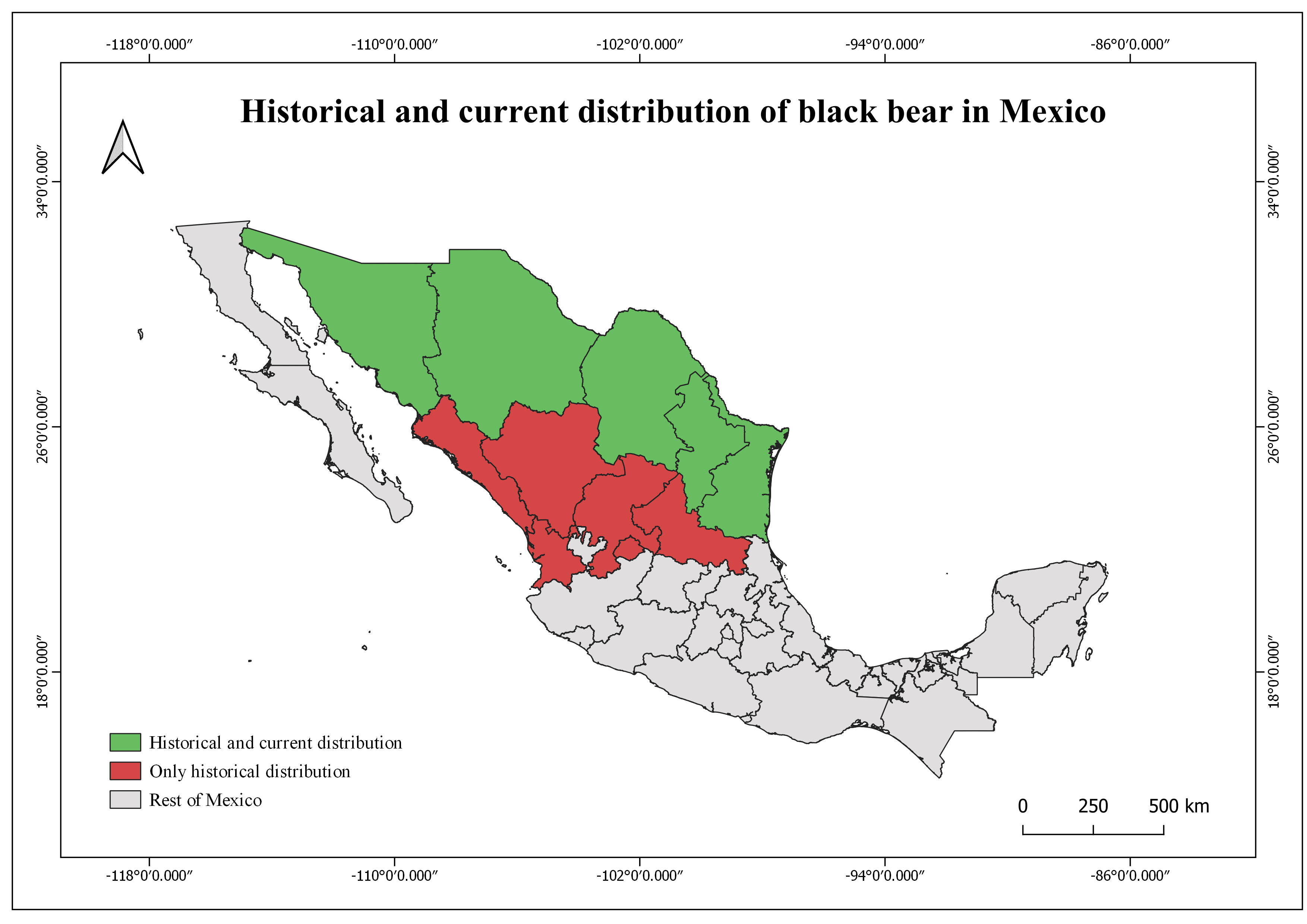

Supplement: S1 Fig — (TIF) [file pone.0272979.s001.tif]
